# RAS-NOTECHS: validity and reliability of a tool for measuring non-technical skills in robotic-assisted surgery settings

**DOI:** 10.1007/s00464-021-08474-2

**Published:** 2021-04-12

**Authors:** Julia Schreyer, Amelie Koch, Annika Herlemann, Armin Becker, Boris Schlenker, Ken Catchpole, Matthias Weigl

**Affiliations:** 1 Institute and Clinic for Occupational, Social and Environmental Medicine, University Hospital, LMU Munich, Ziemssenstrasse 1, 80336 Munich, Germany; 2 Institute for Medical Information Processing, Biometry and Epidemiology (IBE), LMU Munich, Munich, Germany; 3 Department of Urology, University Hospital Grosshadern, Ludwig-Maximilians-University Munich, Munich, Germany; 4 Department of Anesthesia and Perioperative Medicine, Medical University of South Carolina, Charleston, USA; 5 Institute for Patient Safety, University Hospital, Bonn University, Bonn, Germany

**Keywords:** obotic-assisted surgery, Surgical teamwork, Human factors, Non-technical skills, Assessment, Da Vinci system

## Abstract

**Background:**

Non-technical skills (NTS) are essential for safe surgical practice as they impact workflow and patient outcomes. Observational tools to measure operating room (OR) teams’ NTS have been introduced. However, there are none that account for the specific teamwork challenges introduced by robotic-assisted surgery (RAS). We set out to develop and content-validate a tool to assess multidisciplinary NTS in RAS.

**Methodology:**

Stepwise, multi-method procedure. Observations in different surgical departments and a scoping literature review were first used to compile a set of RAS-specific teamwork behaviours. This list was refined and expert validated using a Delphi consensus approach consisting of qualitative interviews and a quantitative survey. Then, RAS-specific behaviours were merged with a well-established assessment tool on OR teamwork (NOTECHS II). Finally, the new tool—RAS-NOTECHS—was applied in standardized observations of real-world procedures to test its reliability (inter-rater agreement via intra-class correlations).

**Results:**

Our scoping review revealed 5242 articles, of which 21 were included based on pre-established inclusion criteria. We elicited 16 RAS-specific behaviours from the literature base. These were synthesized with further 18 behavioural markers (obtained from 12 OR-observations) into a list of 26 behavioural markers. This list was reviewed by seven RAS experts and condensed to 15 expert-validated RAS-specific behavioural markers which were then merged into NOTECHS II. For five observations of urologic RAS procedures (duration: 13 h and 41 min), inter-rater agreement for identification of behavioural markers was strong. Agreement of RAS-NOTECHS scores indicated moderate to strong agreement.

**Conclusions:**

RAS-NOTECHS is the first observational tool for multidisciplinary NTS in RAS. In preliminary application, it has been shown to be reliable. Since RAS is rapidly increasing and challenges for effective and safe teamwork remain at the forefront of quality and safety of surgical care, RAS-NOTECHS may contribute to training and improvement efforts in technology-facilitated surgeries.

In surgery, non-technical skills (NTS) have been shown to be associated with surgeons’ technical performance [1–3], quicker crisis resolution [4], rectification of adverse events [5], operative workflow [6], and objective patient outcomes [3]. NTS are defined as ‘the cognitive, social and personal resource skills that complement technical skills, and contribute to safe and efficient task performance’ (p. 1) [7]. NTS comprise situational awareness, decision-making, leadership, teamwork, and communication [7]. Previous research revealed a relationship between lack of NTS and technical error [2], poor teamwork and operative disruption [8, 9]. Failures in communication are the second most common contributing factor to surgical incidents [10]. One recent study reported that almost a third of intraoperative incidents were deemed avoidable and associated to failures in NTS [11]. Thus, assessment of NTS and efforts to improve surgical teamwork are essential to improve patient safety in the operating room (OR) [11].

Observational tools are commonly used to quantify and evaluate OR teamwork behaviour and NTS [12]. Several direct observational tools have been introduced to identify and evaluate NTS in ORs [13, 14]. The Oxford Non-Technical Skills (NOTECHS) (II) is a well-established tool that has been applied to various surgical specialities [12]. In their systematic reviews, Li et al. and McMullan et al. concluded that NOTECHS is amongst the observational tools with the highest validity and reliability [12, 13]. However, NOTECHS was designed to measure NTS in conventional surgical teamwork settings and may not be applicable to robotic-assisted surgery (RAS).

In the past decade, there has been a tremendous growth in the use of robotic technologies. Yet, RAS adds unique challenges—both technical and non-technical—compared to conventional surgery [15, 16]. During RAS procedures, one surgeon spends much of the operating time on the console, separated from both the patient and the remaining OR team. This requires different strategies of communication and personal interaction among the OR team [17]. While operating, the console surgeon is not able to see other team members’ physical movements and nonverbal answers but has to rely on verbal communication [15]. Other OR team professionals may face challenges with the robot arms obstructing their view of one another. But apart from impeding communication and coordination, RAS also facilitates new modes of nonverbal communication, e.g., with the display as a mediator, providing visual access to all OR team members and allowing for visual communication between the console surgeon and the assistant surgeon [16, 17]. Consequently, available OR teamwork tools may not be sufficiently valid to evaluate NTS in RAS, as they do not take into account RAS-specific behaviours and unique demands [6]. So far, only one observational tool for NTS in RAS has been published: the Interpersonal and Cognitive Assessment for Robotic Surgery (ICARS) [18]. Yet, ICARS merely evaluates NTS of the console surgeon, omitting all other OR professionals’ behaviours, i.e., bedside assistant, anaesthetists, and OR nurses [18]. Taking the key role of multi-professional OR teamwork into consideration, there is a strong need for a tool tailored to RAS that comprehensively evaluates the entire multi-professional OR team [2]. Drawing upon a stepwise systematic procedure, we set out to adapt the NOTECHS specifically to RAS procedures with particular consideration to the challenges introduced by robotic technologies.

## Aim of the study

Specifically, our study aimed for the following objectives:

1. Development of RAS-NOTECHS—an observational tool for the assessment of NTS during RAS that comprises RAS-specific behaviours which are validated by RAS experts, with sub goals of:
  (1a) Synthesis and compilation of RAS-specific teamwork behaviours through the combination of exploratory observations and a scoping review of the current literature base; with subsequent pre-selection of behavioural markers through internal consensus process;
  (1b) Refinement and final selection of RAS behavioural markers through expert validation; synthesis of expert validated behavioural markers with finalization of RAS-NOTECHS.
2. Establishing initial reliability of RAS-NOTECHS in real-world RAS procedures.

## Methods

### Design

We drew up a multi-step procedure to develop a tool for measuring NTS in RAS settings and to test this new tool for content validity and reliability. We used a sequential mixed methods design including direct observations, a scoping review, expert interviews, an expert survey, and structured observations.

The study was approved by the Ethics Committee of the Medical Faculty, Munich University (Nr. 19–696). All participants received written and verbal information prior to data collection. Participation was voluntary and written consent was obtained.

### Description of NOTECHS II

Our work is based on NOTECHS which was originally used in aviation and was adapted for OR teams by Mishra et al. [14]. It has been previously revised to *Oxford NOTECHS II* [19]. NOTECHS II consists of four behavioural dimensions: leadership and management, teamwork and cooperation, problem-solving and decision-making, and situation awareness. For each, there are three to four categories with a description of the generic skills. Positive and negative behavioural markers, specific for each OR subteam (surgery, anaesthesia, nursing), are listed for each dimension [14, 19]. A trained observer rates each OR subteam on the four behavioural dimensions. Scores range from 1 (‘consistently compromises patient safety and effective teamwork’) to 8 (‘consistently enhances patient safety and effective teamwork’) with 6 being the baseline score (‘consistently maintains an effective level of patient safety and teamwork’) [19].

### Procedure

**Fig. 1:**
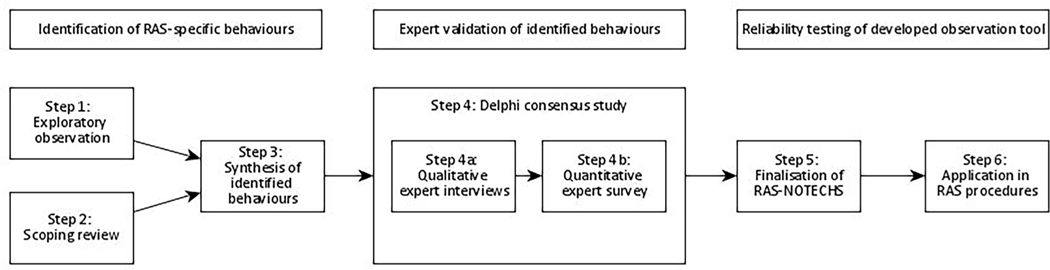
Study procedure flowchart

Our procedure steps are shown in Fig. 1.

### Step 1: Exploratory non-standardized observations to identify RAS-specific teamwork behaviours

In order to obtain a preliminary set of behavioural markers and to gain familiarity with the RAS-setting, we first observed 26 live urologic RAS procedures in a university hospital. Both observers (authors: JS, MW) have a behavioural science background (JS in health sciences, MW in psychology, human factors, and teamwork in acute care and surgical settings). Afterwards, 12 procedures (nine urologic, three visceral surgeries) were observed across different hospital sites (one community and three university hospitals). At all sites, da Vinci surgical systems (Intuitive Surgical, Inc., Sunnyvale, CA, USA, models Si and Xi) were used. Observers took unstructured qualitative notes of what they rated as relevant or RAS-specific behaviours and NTS. They based their notes on the observed behaviours, prior knowledge from preliminary research, and on informal conversations with OR team members of the nursing, anaesthetic, and surgical subteams. From the notes, relevant behaviours were identified in a study team internal review process and extracted into an unstructured list of potentially relevant behavioural markers.

### Step 2: Scoping literature review on RAS-specific teamwork behaviours

In order to identify RAS-specific behavioural markers, we concurrently conducted a scoping review of the literature. Our aim was to elicit NTS or teamwork behaviours that were described as well as which underlying challenges to NTS are introduced into the OR by surgical robotic systems. Our scoping review procedure was based on the guidelines published by the Joanna Briggs Institute [20].

#### Inclusion criteria

Articles were selected based on the following pre-established criteria, shown in Table 1.

#### Search strategy

Articles found in a preliminary search were searched for potential text words (title and abstract), key words, and MeSH-terms. We then searched nine databases (Web of Science, PsycINFO, PubMed, Taylor and Francis, Google Scholar, IEEE Xplore, ACM Digital Library, Science Direct, and Wiley Online Library) for relevant literature. The search strategy consisted of three key concepts: (1) ‘operating room’ or ‘operating room personnel’ (i.e., setting or population), (2) the robotic system (i.e., intervention), and (3) ‘NTS’ (i.e., outcome), the final Pubmed syntax is available (online supplementary file). The search was conducted on April 15 and 16, 2020.

#### Article selection and data extraction of RAS-specific NTS and challenges

First, all duplicate publications were removed. Two reviewers (JS, MW) then independently performed title/abstract screening and full-text screening. After each step, any disagreements were solved by discussion. We further checked reference lists of included publications for additional relevant studies. A data extraction sheet was developed to capture study information, which included first author, year of publication, country of origin, as well as relevant content regarding methodology, surgical specialty, profession, robotic system, RAS-relevant NTS as well as challenges to NTS due to RAS. Data were extracted by the first author (JS).

### Step 3: Synthesis of observation results and scoping review

Retrieved behaviours from steps 1 and 2 were combined into a first comprehensive list of behaviours and, where necessary, rephrased. In a study team’s internal consensus process (JS, AK, MW, KC), we further assigned all behavioural markers to respective NOTECHS II-dimensions and subteams, according to the definitions provided [14, 21]. We excluded behaviours that were not specific (e.g., ‘effective communication is important’ [22]).

### Step 4: Delphi consensus study with OR experts

In order to further condense the list of behaviour markers and to establish content validity, we applied a Delphi consensus approach [23]. The first round consisted of qualitative interviews and the second of a quantitative survey. Participants were OR personnel (three surgeons, three OR nurses, one anaesthetist) from two surgical academic urology departments of large tertiary care hospitals (about 1000 and 2000 hospital beds, respectively). All participants had broad experience with RAS, ranging from 1.5 to 11 years (surgeons between 8.5 and 11 years, OR nurses between 1.5 and 9 years, and anaesthetist 2.5 years).

#### Round one—expert interviews

In order to gather expert views on the behaviours found in literature and observation, we conducted six semi-structured expert interviews discussing the list of behavioural markers (retrieved in step 3). In each interview, the list of behavioural markers was evaluated by an expert who was asked to state whether he/she rated listed behaviours as positive or negative with regard to teamwork, and whether it was relevant to RAS, and to provide justification. Afterwards, experts were asked whether they think of any other, not yet listed teamwork behaviours. Transcripts were based on notes taken throughout the interview, and where possible, audio recordings.

#### Round two—expert survey

Subsequently, interview results (of step 4a) were analysed and the preliminary list of behaviours refined accordingly, i.e., deleting irrelevant items and rephrasing misleading items. This revised list was then utilized for the second round of the Delphi consensus process. The aim of the survey was to find out which behavioural markers were, in the opinion of experts, indicative of good teamwork and would thus be included in RAS-NOTECHS in the next step. Consistent with Hull et al. [24], all experts were asked to rate to which degree each behaviour contributed positively to OR teamwork and patient safety (using a 5-point Likert scale from 1 = ‘not at all’ to 5 = ‘very much’). Both scores for each behavioural marker were combined into a single score (possible range 2–10). Consensus was a priori defined as agreement (combined score 8–10) among > 80% of respondents. In addition to round 1, an additional RAS-urologist took part in round two.

### Step 5: Finalisation of RAS-NOTECHS

By adding the behavioural markers left after the consensus study to the NOTECHS II table of behavioural markers, we created RAS-NOTECHS. NOTECHS II’s dimensions, descriptions of generic skills, and the original behavioural markers were left unchanged [14].

### Step 6: Pilot application in RAS procedures with test for reliability

Finally, RAS-NOTECHS was tested in real-time, standardised observations of RAS procedures in order to assess reliability. In six urologic RAS procedures (one partial nephrectomy, five radical prostatectomies), NTS were rated by two independent observers (JS, MW) simultaneously using the RAS-NOTECHS in order to assess inter-observer reliability. Additionally, a checklist of RAS behavioural markers was filled out in order to find out which of these behaviours were observable. For the observations, the procedures were divided into several phases [‘*wheels in* to *insufflation*’, ‘*insufflation* to *surgeon at console*’, ‘*surgeon at console* to *surgeon off console*’ (further divided into 20 min intervals), ‘*surgeon off console* to *closure*’]. RAS-NOTECHS ratings and the behaviour checklist were assessed for each phase, respectively. Intra-class correlation (ICC) estimates with 95% confidence intervals were calculated for the overall RAS-NOTECHS rating as well as for each RAS-NOTECHS dimension (with a single measure, absolute agreement, two-way mixed-effects model). We calculated ICCs for the overall as well as of each RAS-NOTECHS dimension, respectively. Following the definition by Koo, Li [25], ICC values < 0.5, 0.5–0.75, 0.75–0.9, and > 0.9 are indicative of poor, moderate, good, and excellent reliability, respectively. For agreement on individual RAS behaviours, Gwet’s AC1 [26] was calculated. Descriptive analyses were conducted to determine how frequently behavioural markers were observed. We used SPSS 25 (IBM Inc., Chicago).

## Results

### Step 1: Exploratory observation results

After 73 h and 51 min of observer training, we observed a total of 39 h and 51 min of RAS procedures (radical prostatectomies, partial nephrectomies, adrenalectomies, pyeloplasties, sigmoid resections, intestinal resections) using the da Vinci surgical system. 25 observed behaviours were extracted from observation notes and condensed into a preliminary collection of 18 behavioural markers deemed to be relevant to RAS. This preliminary list is available upon request.

### Step 2: Scoping review results

Our systematic literature search retrieved 5242 articles. After duplicate removal and abstract screening, 57 articles underwent full-text review. Finally, 21 eligible articles were included for data extraction, elicitation and synthesis of RAS-specific behaviours.

#### Characteristics of included articles

Table A1 of the online supplementary files shows characteristics of the 21 included articles. All were published between 2004 and 2020 (median: 2017). Eleven studies were conducted in the US, [15, 17, 27–35], six in the UK [18, 36–40], and four in Western/Northern European countries [22, 41–43]. Studies either examined one [18, 22, 28, 32, 41] or several OR professions [15, 17, 27, 29–31, 33–40, 42, 43]. The majority investigated only one surgical specialty [17, 18, 27–30, 33–35, 38]. Eight studies focused on da Vinci surgical systems (Intuitive Inc., CA) [18, 27, 36, 37, 40–43], one examined Brock Rogers Surgical Laprotek System [29].

#### Challenges to NTS and team behaviours in RAS settings

Results of the scoping review concerning challenges to NTS that are introduced by RAS are shown in Table 2. Sixteen NTS or teamwork behaviours were extracted. Most of these were mentioned in several studies (e.g., ‘use of explicit communication’ [15, 18, 31, 37, 41, 42]). Fewer of them were reported only in a single publication (e.g., ‘surgeon enforces read-back if no confirmation is produced’ [41]).

### Step 3: Synthesis results

After both preliminary lists from step 1 and step 2 were combined, the collated list of RAS behavioural markers consisted of 26 items. This list, including its respective NOTECHS dimension, the OR subgroup it refers to, as well as behavioural examples from literature or observation, was used to develop an interview guide for the following expert interviews (this preliminary list can be obtained from the authors upon request).

### Step 4: Delphi consensus study results

#### Round one—interview results

All experts deemed the list of behaviours comprehensive. Some behaviours were rephrased, and one behavioural marker was split in two (‘console surgeon keeps team engaged by commenting on operative steps, providing educational commentary’ was changed to ‘console surgeon keeps team engaged by commenting on operative steps’ and ‘console surgeon keeps team engaged by conversation’, as experts said that unrelated conversations also helped stay engaged). Six behaviours were excluded from the list because the experts either agreed that this behaviour was neither negative nor positive, or that this behaviour was not specific to RAS. After expert interviews, the list comprised 21 behavioural markers. These were then converted into round two’s survey.

#### Round two—survey results

Pre-defined consensus was reached for 15 of the initial 21 survey items. On seven of these, experts agreed 100%.

### Step 5: Finalisation of RAS-NOTECHS results

Finally, 15 expert-approved behavioural markers were added to NOTECHS II (p. 6, table of subteam modifiers) [14]. Twelve behavioural markers were assigned to surgeons, five to anaesthesia, and nine to OR nursing (some were also assigned for two or three professions). Four were added to the dimension *leadership and management*, five (three for all subgroups, two additional for nursing subgroup) to *teamwork and cooperation*, one to *problem-solving and decision-making*, and five to *situation awareness*. The final RAS-NOTECHS including the table of behavioural markers is presented in the online supplement (Tables A2–A4).

### Step 6: Descriptive analysis results and inter-rater agreement results

The first pair-wise observation served as a calibration session, after which some small adjustments to RAS-NOTECHS were made. Following this, two trained observers (JS, MW) simultaneously observed 13 h and 41 min of RAS procedures (convenience sample of one partial nephrectomy, four radical prostatectomies; all performed with da Vinci model Xi).

**Fig. 2:**
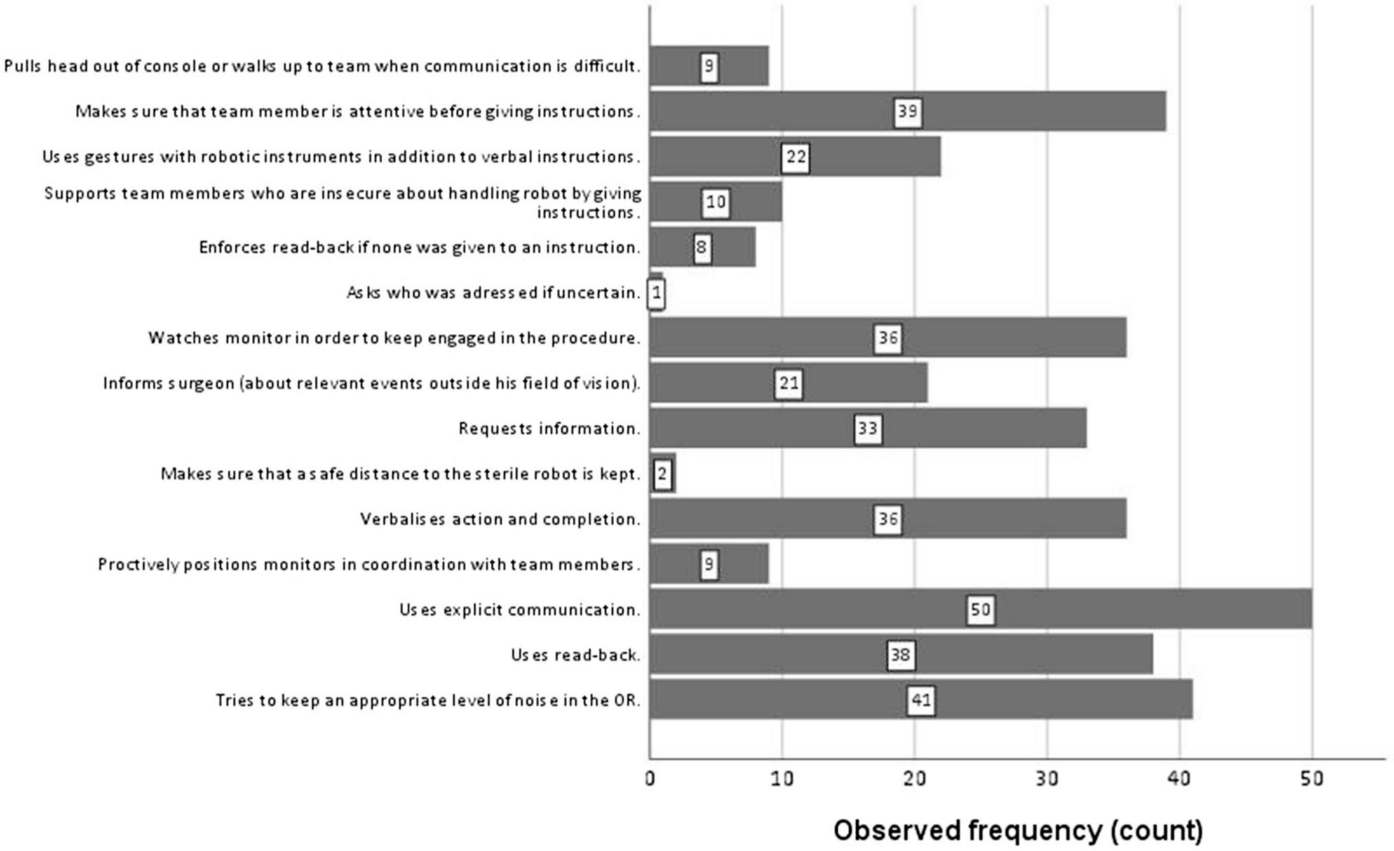
Observed frequency of RAS-specific behaviours (overall *n* = 52 observation phases)

As shown in Fig. 2, some behaviours such as ‘uses explicit communication’ and ‘tries to keep an appropriate level of noise in the OR’ were very prevalent (96.2% and 78.9%, respectively). Others such as ‘makes sure that a safe distance to the sterile robot is kept’ (3.8%), ‘proactively positions monitors in coordination with team members’ (17.3%) or ‘asks who was addressed if uncertain’ (1.9%) were rarely observed. The online supplement (Table A5) lists examples for each behaviour.

A moderate degree of correlation was found for the scores of the complete RAS-NOTECHS (ICC 0.687, 95% CI [0.639; 0.729]) as well as for scores of RAS-NOTECHS dimensions *leadership and management* (ICC 0.690, 95% CI [0.583; 0.773]) and *situation awareness* (ICC 0.500, 95% CI [0.360; 0.618]). For the dimensions *teamwork and cooperation* and *problem-solving and decision-making,* we achieved good agreement (ICC 0.812, 95% CI [0.745; 0.863], ICC 0.789, 95% CI [0.715; 0.846], respectively). For inter-rater agreement for the list of individual behaviours we obtained a Gwet’s AC1 of 0.831, 95% CI [0.789, 0.874], indicating strong agreement [44, 45].

## Discussion

The aim of this study was to develop a tool for measuring teamwork of RAS teams as well as to test its reliability. A systematic, stepwise and mixed-methods procedure was applied. RAS-NOTECHS encompasses relevant behavioural markers for effective OR teamwork in RAS. Thus, our study results contribute to the current literature base in several ways:

First, we introduce a new observational tool that allows assessment of teamwork behaviours in RAS. Robot-facilitated surgery is rapidly growing and surgical technology is advancing with an increasing demand and use across different procedures [46–48]. Since OR teamwork behaviours are an important factor in safety and quality of delivery of surgical care [2, 3, 5, 11], a reliable and valid tool for assessing NTS in RAS was needed to evaluate multi-professional teamwork in RAS. RAS-NOTECHS is expert validated and has been shown to be a reliable instrument.

Second, drawing upon a scoping review of the current scientific literature base on teamwork in RAS, we systematically identified a comprehensive set of behaviours that are deemed critical for successful collaboration in RAS. Further, this extended set of behaviours was evaluated by clinicians from surgery, nursing, and anaesthesia. The result of the consensus process is a validated set of RAS-critical behaviours. It comprises teamwork behaviours that are assessable, tangible and can be utilized for training, simulation, and teamwork improvement measures [13, 49–52].

Our obtained behavioural markers are to some extent similar to behaviours OR teams show in conventional surgical settings (e.g., open surgery). Yet, we deem that these behaviours are more critical in robotic settings as they may compensate for the unique challenges to NTS and teamwork introduced by RAS [15, 37]. Additionally, most of the identified behavioural markers either applied to the console surgeon or bedside team alone, or the entire team. Hence, there was no new behavioural marker that exclusively applied to the anaesthesia subteam. This finding is consistent with our scoping review findings: the robot presents challenges mainly for the surgical and nursing subteams by creating physical separation and changing their task load [36–38]. Spatial separation between the anaesthesia subteam and the operating table is also applicable to conventional OR set-ups. However, RAS-specific anaesthesiologic demands remain, e.g., with regard to high relaxation needs or difficult patient positioning, such as Trendelenburg [28].

Despite our focus on potential RAS-related barriers and obstacles to NTS as we elicited in Table 2, it is important to note that there are also relevant teamwork advantages facilitated through RAS. For example, the console surgeon and assistant surgeon can communicate via the screen either using their instruments to point at important areas in situ [15] or by using telestration [53]. Additionally, as each OR team member is able to observe the progression of the surgery on the screen, the team’s shared situation awareness increases [16].

While we aimed to identify behaviours indicating good or poor teamwork, we found that some were only applicable if a negative behaviour or event preceded, i.e., actually compensated for obstacles of the robotic setup [15, 35, 37, 40]: for example, ‘surgeon pulls head out of console or walks up to operating table if communication with team is difficult’ or ‘OR team member asks if uncertain who was addressed’ can be considered as a necessary rectification of a suboptimal condition. Since we consider OR professional’s adequate and effective adaption to evolving challenges an important NTS, these behavioural markers were kept.

We found RAS-NOTECHS to be a reliable teamwork assessment tool throughout the first applications in real-world procedures. Inter-rater agreement was at least moderate in all dimensions which is similar to other studies using NTS observational tools in surgery [19, 49]. Our inter-rater agreement of the individual behavioural markers was strong. The variance of observed behaviours is not surprising: particular behaviours are general and apply to almost each step of a surgery (e.g., ‘uses explicit communication’), others are only necessary either in specific phases of a procedure or as compensation for a negative circumstance (e.g., ‘proactively positions monitors in coordination with team members’, ‘asks who was addressed if uncertain’). Future research should confirm reliability and feasibility across different surgical settings, procedures, as well as test for robustness across various observers’ backgrounds [54]. In our study, RAS-NOTECHS was content-validated by RAS experts. We did not test for construct nor criterion validity. As these kinds of validity testing are important in order to establish overall validity of RAS-NOTECHS, we recommend assessing (1) the relationship with other NTS measures and (2) the relationship between RAS-NOTECHS scores and relevant safety and patient outcomes, e.g., number of near-miss events [55].

Third, given the key role of NTS in technology-facilitated surgery and the high pace of technological innovations in surgery, we deem that our multi-step approach may serve as a blueprint for similar attempts to capture teamwork behaviours in high-technology care settings. Given the increasing role of new technology in the OR, multi-disciplinary and inter-professional teamwork will remain crucial for a safe and efficient delivery of surgical care [56, 57]. We propose a systematic procedure for the development of observational tools, combining the current literature base, expert knowledge, and real-life observations.

### Limitations

Our study has some limitations. First, we conducted our observations (step 1 and 6) in four university or teaching hospitals in Germany, all working with da Vinci surgical systems. This may limit our proposed behaviours, as possible differences between countries or across robotic systems were not identified. Notwithstanding, our scoping review accounted for variety in RAS practice as the included articles originated from various countries. Just one article specified dealing with a different robotic system, which was, however, similar in setup and resulting challenges [29]. The da Vinci surgical system is currently by far the most widely implemented RAS system worldwide [29], and other surgical systems that are being introduced to the market share its basic setup (e.g., surgeon at console being physically separated) [58, 59]. Therefore, even though RAS-NOTECHS behavioural markers were mainly based on observations and literature on the da Vinci surgical system, they are highly likely to apply to RAS using other surgical systems.

Second, bias inherent to observational studies may have occurred, i.e., participant reactivity and observer bias [60]. Also, since we only observed urologic procedures in one institution for reliability testings (step 6), further tests across specialties and in different institutions are advised.

Third, for expert validation we used a convenience sample of OR staff from only two surgical departments. Our sample size of experts may incur bias concerning institutional practices that are idiosyncratic to specific departments. Our geographically restricted sample of experts and imbalance across professions may have influenced the results of our Delphi consensus study. However, the opinions and behaviours of our experts are in line with the results of our literature review. Future research should investigate how teamwork behaviours in RAS depend upon set-ups (i.e., position of console in relation to anaesthetist and assistant, layout of OR).

Last, we may have possibly missed some relevant literature in our scoping review (i.e., exclusion of grey literature, language restrictions). However, most of the extracted behaviours were consistently mentioned in several of included publications. Additionally, all interviewed expert clinicians deemed the set of behaviours comprehensive. Hence, we assume that all relevant behaviours have been identified. Our scoping review is a first step in examining the literature base of specific NTS in RAS. In the future, methodological quality of included studies may be evaluated and taken into account. In fact, a previous systematic review on a topic comparable to ours concluded that the available literature base on NTS within RAS has methodological limitations [16].

### Implications

With regard to implications for future research, RAS-NOTECHS can be applied during observation of real-life procedures and correlated with procedure or patient outcomes in order to expand the current knowledge about the effect of NTS on quality of healthcare delivery. RAS-NOTECHS may assist in assessing effects of teamwork trainings by comparing RAS-NOTECHS scores before and after a training session (e.g., through educational interventions, simulations [61]). In healthcare settings other than RAS, NTS training has been shown to improve NTS performance during simulation or in real-life care [52, 62, 63] and was associated with improvements in patient outcomes [63].

Future research should consider potential influences on RAS teamwork that we did not specifically address in the development and validation of RAS-NOTECHS, such as low- vs. high-complexity procedures, team familiarity (i.e., prior experience of working together, fixed RAS teams), and dealing with intraoperative events or disruptions [64].

With regard to implications for surgical practice, the set of RAS-specific behaviours itself can serve as a basis for teaching teamwork in RAS. We found that OR staff already perform most of the identified behaviours in open surgery, but to a lesser extent compared to RAS, implicating that almost no new skills need to be taught in RAS-NTS training. In fact, our findings advocate that existent skills need to be considered and targeted in RAS-training and education. To this end, already existing and applied training approaches might be adapted [62, 65, 66].

Our new knowledge might be taken into account for the future design of robotic surgical technologies in order to facilitate intraoperative teamwork. We found that many RAS-specific behaviours are necessary to compensate for challenges introduced by the surgical robot, such as obstructed views between console surgeon and the rest of team, bulky equipment [35], difficulties of verbal communication, or low situation awareness [37]. By applying human-centred design concepts, developers should consider how they can prevent behaviours that compensate for suboptimal conditions and how to design robotic technologies that foster smooth collaboration within the OR team.

## Conclusions

In our study, we introduce RAS-NOTECHS—the first behavioural rating system for multidisciplinary NTS in RAS. We deployed a stepwise mixed-methods approach combining the current evidence base, expert knowledge, and real-life observations. RAS-NOTECHS is reliable and can be used to identify RAS-specific behavioural markers in real-life procedures. Our study provides assessment methodologies for future research investigating the role of NTS for safety and quality of delivery of surgical care in technology-facilitated teamwork.

## Supplementary Material

Supplementary Material

## Figures and Tables

**Table 1:** Inclusion and exclusion criteria for article selection

Inclusion criteria	Exclusion criteria

Topic: non-technical skills or team behaviours in RAS-setting	Studies on…
Scientific article of any kind (original study, review, letter, reports)	Technical development of surgical robots or specific parts of surgical robots (technical challenges, designs, …)
Setting: surgeries facilitated by robotic technology	Clinical applicability of surgical robots
Time frame: Articles published between 1995 and 2020	Aetiology/pathology of a disease
Language: English or German	Therapy options other than surgery (such as medication)
	Surgical therapy options for specific diseases (options, outcomes, feasibility, clinical trials)
	Robots other than surgical robots (robotic nurse, therapy)
	Telemedicine

**Table 2:** Challenges to NTS during RAS synthesized from scientific literature (including references)

Reason	Challenge

Change in task distribution	Additional tasks for surgeon: responsible for distributing more information to surgical team (e.g., changing instruments) [1]
Physical separation of console surgeon and rest of team	Surgeon can control more instruments; Reduction in task load for bedside team (scrub practitioner, first assistant), this can lead to decreased engagement in procedure and awareness of processes, making the team less responsive to the console surgeon’s need for assistance [2–4] Harder for team to hear surgeon’s request, communication has to be repeated often (+ / microphone bad) (esp. when immersed) [2, 4–9] Unclear who surgeon is talking to [2]
	Surgeon cannot see operating table, relies on surgical assistant and scrub team to communicate [2, 3,10–12]
	Surgeon cannot see patient, depends on team to inform him, increased coordination and communication demands [2, 7,12]
	Surgeon cannot see robot, depends on team to inform him [1, 2]
	Surgeon is immersed in console, cannot see team (e.g., no visual feedback that message has been received, needs verbal confirmation that request was accepted and fulfilled) [6–8,11,12]
	Surgeon unscrubbed, not at table bedside team has to respond to complication, needs higher shared awareness [2]
	Immersed in console, tunnel vision, less aware of what others are doing [2,3,8,10,13,14]
	Team cannot see surgeon difficult for team to monitor surgeon’s actions and facial cues [2–4,12,13]
	Team members, especially console surgeon, can experience a sense of isolation [7,8,13]
	Harder to interpret directional cues [1, 2, 4,15,16]
	Surgeon and team cannot see each other, cannot communicate via gestures, movements, face to face communication [9,17–19]
	Impedes face to face implicit control, Non-verbal communication difficult, reliance on verbal exchanges, changed feedback-loop [1]
Robotic system itself	Robot/additional equipment size space constraints [2,5, 9,10]
	Robotic system: no tactile information [1, 2, 7,19] Communication via bidirectional device staff-side talks distracting to console surgeon [8]
