# Supplementary material for: RAS-NOTECHS: validity and reliability of a tool for measuring non-technical skills in robotic-assisted surgery settings
Source: Surg Endosc. Author manuscript; Available in PMC 2022 Mar 1. (PMC8505574; doi:10.1007/s00464-021-08474-2)
Supplement: Supplementary file 1 [file NIHMS1697928-supplement-Supplementary_Material.docx]

**Online supplementary files**		

**RAS-NOTECHS Scoping Review: PubMed syntax**

(anaesthetist*[tiab] OR anesthetist*[tiab] OR anaesthesiologist*[tiab] OR anesthesiologist*[tiab] OR intraoperative[tiab] OR nurse[tiab] OR nurses[tiab] OR nurse’s[tiab] OR nurses’[tiab] OR "operating room"[tiab] OR "operating theatre"[tiab] OR "operative setting"[tiab] OR perioperative[tiab] OR surgeon[tiab] OR surgeons[tiab] OR surgeon’s[tiab] OR surgeons’[tiab] OR surgery[tiab] OR surgical[tiab] OR “surgical team” [tiab] OR “surgical teams” [tiab] OR “team members” [tiab] OR “team member” [tiab]  OR “operating room team” [tiab] OR “operating room teams” [tiab] OR operating room[MeSH Terms] OR operating room technicians[MeSH Terms] OR operating room nursing[MeSH Terms] OR intraoperative period[MeSH Terms] OR intraoperative care[MeSH Terms] OR surgeon[MeSH Terms] OR patient care team[MeSH Terms])

AND (ras[tiab] OR “robotic surgery” [tiab] OR “robot-assisted surgery” [tiab] OR “robotic assisted surgery” [tiab] OR “robotic-assisted surgery” [tiab] OR “surgical robot” [tiab] OR “da vinci” [tiab] OR robot-facilitated[tiab] OR “robot system”[tiab] OR console[tiab] OR robotic surgery[MeSH Terms]) 

AND (nts[tiab] OR “non-technical skills” [tiab] OR “non technical skills” [tiab] OR “nontechnical skills” [tiab] OR communicat*[tiab] OR coordinat*[tiab] OR cooperat*[tiab] OR collaborat*[tiab] OR “decision making” [tiab] OR cognition[tiab] OR teamwork[tiab] OR “team work” [tiab] OR team process*[tiab] OR “situation awareness” [tiab] OR leadership[tiab] OR interpersonal skill*[tiab] OR behaviour*[tiab] OR behavior*[tiab] OR distraction[tiab] OR concentration[tiab] OR “mental focus” [tiab] OR “information sharing” [tiab] OR stress[tiab] OR communication[MeSH Terms] OR nonverbal communication[MeSH Terms] OR communication barriers[MeSH Terms] OR interpersonal relations[MeSH Terms] OR decision making[MeSH Terms] OR Crew Resource Management, Healthcare[MeSH Terms] OR Awareness[MeSH Terms] OR social skills[MeSH Terms] OR group processes[MeSH Terms])

AND ("01/01/1995"[Date - Publication] : "3000"[Date - Publication])

Note: Syntaxes for the other databases are available upon request.

**Table A1** Characteristics of articles included in scoping review

| **Authors** | **Year** | **Country** | **Study type** | **Profession(s)** | **Surgical specialty** | **Robotic system** |
| --- | --- | --- | --- | --- | --- | --- |
| A. Gill, R. Randell [1] | 2016 | UK | Literature review | NA | NA | NA |
| A. Nyssen, A. Blavier [2] | 2009 | BE | Observational study | Uniprofessional | Several | da Vinci |
| A. Satchidanand et al. [3] | 2019 | USA | Observational study | Multiprofessional | One | NA |
| A. Sergeeva et al. [4] | 2015 | NL | Observational and interview study | Multiprofessional | NA | da Vinci |
| C. Almeras, C. Almeras [5] | 2019 | FR | Cross-sectional survey | Multiprofessional | Several | da Vinci |
| C. Cao, H. Taylor [6] | 2004 | USA | Observational study | Uniprofessional | One | LaproTek |
| D. El-Hamamsy et al. [7] | 2020 | UK | Observational and interview study | Multiprofessional | Several | da Vinci |
| F. Lai, E. Entin [8] | 2005 | USA | Interview study | Multiprofessional | NA | NA |
| H. Pelikan [9] | 2018 | USA/NL | Observational study | Multiprofessional | One | da Vinci |
| J. Tiferes et al. [10] | 2016 | USA | Observational study | Multiprofessional | NA | NA |
| K. Catchpole et al. [11] | 2019 | USA | Literature review | Multiprofessional | Several | NA |
| K. Wastler [12] | 2015 | USA | Short communication | Uniprofessional | NA | NA |
| M. Myklebust et al. [13] | 2020 | NOR | Interview study | Uniprofessional | NA | NA |
| N. Alvarado et al. [14] | 2017 | UK | Literature review and interview study | Multiprofessional | One | NA |
| N. Raison et al. [15] | 2017 | UK | Multi-method study | Uniprofessional | One | da Vinci |
| R. Hsu et al. [16] | 2013 | USA | Literature review | NA | One | NA |
| R. Randell et al. [17] | 2017 | UK | Multi-method study | Multiprofessional | Several | da Vinci |
| R. Randell et al. [18] | 2019 | UK | Multi-method study | Multiprofessional | Several | da Vinci |
| S. Raheem et al. [19] | 2018 | USA | Observational study | Multiprofessional | One | NA |
| T. Higuchi, M. Gettman [20] | 2011 | USA | Book chapter | Multiprofessional | NA | NA |
| T. Payne, M. Pitter [21] | 2011 | USA | Literature review | NA | One | NA |
| Note: NA = not applicable or not available |  |  |  |  |  |  |

**Appendix Table A2 through A4** **Complete RAS-NOTECHS**

**Table A2** Operating-theatre team Non-Technical Skills (NOTECHS) assessment tool

| **Leadership and management** |  |
| --- | --- |
| Leadership | Involves/reflects on suggestions/visible/accessible/inspires/motivates/coaches |
| Maintenance of standards | Subscribes to standards/monitors compliance to standards/intervenes if deviation/deviates with team approval/ demonstrates desire to achieve high standards |
| Planning and preparation | Team participation in planning/plan is shared/understanding confirmed/projects/changes in consultation |
| Workload management | Distributes tasks/monitors/reviews/tasks are prioritised/allots adequate time/responds to stress |
| Authority and assertiveness | Advocates position/values team input/takes control/persistent/appropriate assertiveness |
| **Teamwork and cooperation** |  |
| Team building/maintaining | Relaxed/supportive/open/inclusive/polite/friendly/use of humour/does not compete |
| Support of others | Helps others/offers assistance/gives feedback |
| Understanding team needs | Listens to others/recognises ability of team/condition of others considered/gives personal feedback |
| Conflict solving | Keeps calm in conflicts/suggests conflict solutions/concentrates on what is right |
| **Problem-solving and decision-making** |  |
| Definition and diagnosis | Uses all resources/analytical decision-making/reviews factors with team |
| Option generation | Suggests alternative options/asks for options/reviews outcomes/confirms options |
| Risk assessment | Estimates risks/considers risk in terms of team capabilities/estimates patient outcome |
| Outcome review | Reviews outcomes/reviews new options/objective, constructive and timely reviews/makes time for review/seeks feedback from others/conducts post-treatment review |
| **Situation awareness** |  |
| Notice | Considers all team elements/asks for or shares information/aware of available of resources/encourages vigilance/ checks and reports changes in team/requests reports/updates |
| Understand | Knows capabilities/cross-checks above/shares mental models/speaks up when unsure/updates other team members/discusses team constraints |
| Think ahead | Identifies future problems/discusses contingencies/anticipates requirements |
| Note: source: Mishra et al. 2009 [22] |  |

**Table A3** Behavioural parameters of Oxford NOTECHS II

| **Behaviour** | **Frequency** | **RAS-NOTECHS score** |
| --- | --- | --- |
| Compromises patient safety and effective teamwork | Consistently | 1 |
|  | Inconsistently | 2 |
| Could directly compromise patient safety and effective teamwork | Consistently | 3 |
|  | Inconsistently | 4 |
| Maintains an effective level of patient safety and teamwork | Inconsistently | 5 |
|  | Consistently | 6 |
| Enhances patient safety and effective teamwork | Inconsistently | 7 |
|  | Consistently | 8 |
| Note: source: Robertson et al. 2014 [23] |  |  |

**Table A4** RAS-NOTECHS subteam modifiers

|  | **Surgical subteam** | **Anaesthetic subteam** | **Nursing subteam** |
| --- | --- | --- | --- |
| **Leadership and management** |  |  |  |
| Positive modifiers | Raises team morale Intervenes if deviation Prioritises tasks Pulls head out of console or walks up to operating table when communication with team is difficult Makes sure that team member is attentive before giving instructions Uses gestures with robotic instruments in addition to verbal instructions Supports team members who are insecure about handling robot by giving instructions | Takes control when required Demonstrates desire for high standard Appropriately distributes tasks b/w rest of team | Scrub nurse provides clear instructions to circulating nurse(s) Senior nurse makes sure protocols are followed Speaks up when unhappy |
| Negative modifiers | Deflates or fails to motivate team | Does not set standards Inappropriate task distribution | Senior nurse does not support juniors |
| **Teamwork and cooperation** |  |  |  |
| Positive modifiers | Open Appropriate use of abilities within team Supportive of other subteams when necessary Uses explicit communication Uses read-back Tries to keep an appropriate level of noise in the OR | Supportive of other subteams Appreciates function of other subteams Tries to keep an appropriate level of noise in the OR Uses read-back Uses explicit communication | Nurses cooperate and support each other well Senior nurse covers for junior scrub verbalises action and completion Tries to keep an appropriate level of noise in the OR Uses read-back Uses explicit communication Circulating nurse proactively positions monitors in coordination with team members |
| Negative modifiers | Aggressive in conflicts Does not appreciate others‘ abilities | Remains idle when problems arise Functions separately from other subteams | Poor coordination between equipment needs and those provided |
| **Problem-solving and decision-making** |  |  |  |
| Positive modifiers | Demonstrates generation of options Open discussion and agreement over anatomy Incorporates other subteam issues Console surgeon enforces read-back if none was given to an instruction | Participates in solving problems Raises suggestions | Takes an active part in decision-making Suggests solutions to problems – e.g., alternative equipment |
| Negative modifiers | Decisions made unsystematically Does not utilise team where it may benefit | Does not consider anaesthetic options when met with problem | Blames the surgeon when faced with problems |
| **Situation awareness** |  |  |  |
| Positive modifiers | Periodically gathers awareness of surroundings Asks who was addressed if uncertain Watches monitor in order to keep engaged in the procedure Informs console surgeon (about relevant events outside his field of vision) Requests information | Anticipates surgical and process needs Asks who was addressed if uncertain Watches monitor in order to keep engaged in the procedure | Anticipates equipment needs informs console surgeon (about relevant events outside his field of vision) Makes sure that a safe distance to the sterile robot is kept Asks who was addressed if uncertain Watches monitor in order to keep engaged in the procedure |
| Negative modifiers | Is fixated on operative field | Is not present at important stages of the operation or for long periods of time | Absent at stages when needed to provide service |
| For all subteams Positive modifiers | Patient: has awareness of patient condition/comorbidity Procedure: appreciates stage of operation People: who is present in theatre, what skills they have and what they are doing |  |  |
| Note: adapted from Mishra et al. 2009 [22] |  |  |  |

**Appendix Table A5 Observation examples for behavioural markers**

| **Dimension** | **Behavioural marker** | **Example from observations** |
| --- | --- | --- |
| Leadership and management | Console surgeon pulls head out of console or walks up to operating table when communication with team is difficult | Console surgeon leaves console to check pressure (circulating nurse not in OR) Before critical phase, surgeon turns away from console and towards team to discuss the next steps |
|  | Console surgeon makes sure that team member is attentive before giving instructions | Starts instruction by saying first name of circulating nurse (and pausing for a short moment before further instructions) |
|  | Console surgeon uses gestures with robotic instruments in addition to verbal instructions | Console surgeon says “clip here” (to assisting surgeon) and confirms location of clip placement by indicating vessel or structure with robotic instrument, i.e., force bipolar |
|  | Console surgeon supports team members who are insecure about handling robot by giving instructions | Surgeon gives clear instructions to circulating nurse (who is not familiar with the robot) while nurse is navigating the robot |
| Problem-solving and decision-making | Console surgeon enforces read-back if none was given to an instruction | Console surgeon to scrub nurse, ‘[name], did you hear that I told you to unblock the catheter?’ Console surgeon repeats instruction |
| Situation awareness | Team members watch monitor in order to keep engaged in the procedure | Circulating nurse stands close to the monitor in order to easily follow the surgical steps Anaesthetist stands up and watches the monitor |
|  | Bedside team inform console surgeon (about relevant events outside his field of vision) | Console surgeon: ‚Please suck the urine out of the bladder.’, assistant ‘We’ve just done it.’ Assistant tells surgeon that sucker is malfunctioning and he is not able to suck at the moment |
|  | Nurse makes sure that a safe distance to the sterile robot is kept | During patient positioning, scrub nurse stands next to sterile robot to ensure that no one accidentally gets too close to the sterile field |
|  | Team members ask who was addressed if uncertain | Surgeon asks for Trendelenburg position of the table, which is usually performed by the anaesthetist. Anaesthetist not familiar with this and asks surgeon ‘Do you usually do that or are you speaking to me?’ |
|  | Console surgeon requests information | Asks whether robot arm is blocked Asks ‘catheter has been removed, right?’ ‘Have you already prepared a drain?’ |
| Teamwork and cooperation | Nurse verbalises action and completion | ‚Increase pressure to 20 … pressure is 20’ ‚Catheter is fixed’ ‘Needle is out’ ‚OR table is moving’ |
|  | Team members use explicit communication | Console surgeon: ‘please take the catheter and insert it now’ Console surgeon ‘please clean the camera’ ‘turn OR light on’ |
|  | Team members use read-back | Scrub nurse answers to console surgeon ‘I’m inserting the catheter now’ |
|  | Team members try to keep an appropriate level of noise in the OR | Anaesthetist and nurse anaesthetist whisper |
|  | Circulating nurse proactively positions monitors in coordination with team members | After docking of the robot, the circulating nurse moves monitor so bedside surgeons are able to see it |

**References of tables**

1. Gill A, Randell R. Robotic surgery and its impact on teamwork in the operating theatre. J Perioper Pract. 2016;26(3):42-5. doi: 10.1177/175045891602600303.

2. Nyssen A-S, Blavier A. Verbal communication as a sign of adaptation in socio-technical systems: the case of robotic surgery. 9th Bi-annual International Conference on Naturalistic Decision Making (NDM9) 9. 2009:267-72. doi: 10.14236/ewic/NDM2009.39 

3. Satchidanand A, Higginbotham J, Bisantz A, Aldhaam NA, Elsayed AS, Kannappan R, et al. Vague swirling ends in no communication: referencing strategies during robot assisted surgery. J Urol. 2019;201(4):E288-E9. 

4. Sergeeva A, Huysman M, Faraj S. Transforming work practices of operating room teams: the case of the Da Vinci robot.  Transforming surgery practice with the Da Vinci robot Thirty Sixth International Conference on Information Systems. Fort Worth 2015.

5. Almeras C. Operating room communication in robotic surgery: Place, modalities and evolution of a safe system of interaction. J Visc Surg. 2019;156(5):397-403. doi: 10.1016/j.jviscsurg.2019.02.004.

6. Cao CG, Taylor H. Effects of new technology on the operating room team. Tufts Univ Medford MA Dept of Mechanical Engineering; 2004. p. 309-12.

7. El-Hamamsy D, Walton TJ, Griffiths TRL, Anderson ES, Tincello DG. Surgeon-Team Separation in Robotic Theaters: A Qualitative Observational and Interview Study. Female Pelvic Med Reconstr Surg. 2020;26(2):86-91. doi: 10.1097/spv.0000000000000829.

8. Lai F, Entin E. Robotic surgery and the operating room team. Proceedings of the human factors and ergonomics society annual meeting. 2005;49(11):1070-3. doi: 10.1177/154193120504901115.

9. Pelikan HR. " What's going on there?" Negotiating common ground in robotic vs. open Surgery: a comparison of surgeon-initiated requests for action in open and robotic surgery. University of Twente; 2018.

10. Tiferes J, Hussein AA, Bisantz A, Kozlowski JD, Sharif MA, Winder NM, et al. The Loud Surgeon Behind the Console: Understanding Team Activities During Robot-Assisted Surgery. J Surg Educ. 2016;73(3):504-12. doi: 10.1016/j.jsurg.2015.12.009.

11. Catchpole K, Bisantz A, Hallbeck MS, Weigl M, Randell R, Kossack M, et al. Human factors in robotic assisted surgery: Lessons from studies 'in the Wild'. Appl Ergon. 2019;78:270-6. doi: 10.1016/j.apergo.2018.02.011.

12. Wastler KE. Robotic surgical and anesthesia communication tool. J Robot Surg. 2015;9(1):97-8. doi: 10.1007/s11701-014-0494-8.

13. Myklebust MV, Storheim H, Hartvik M, Dysvik E. Anesthesia Professionals' Perspectives of Teamwork During Robotic-Assisted Surgery. Aorn J. 2020;111(1):87-96. doi: 10.1002/aorn.12897.

14. Alvarado N, Honey S, Greenhalgh J, Pearman A, Dowding D, Cope A, et al. Eliciting context-mechanism-outcome configurations: Experiences from a realist evaluation investigating the impact of robotic surgery on teamwork in the operating theatre. Evaluation. 2017;23(4):444-62. doi: 10.1177/1356389017732172.

15. Raison N, Wood T, Brunckhorst O, Abe T, Ross T, Challacombe B, et al. Development and validation of a tool for non-technical skills evaluation in robotic surgery-the ICARS system. Surg Endosc. 2017;31(12):5403-10. doi: 10.1007/s00464-017-5622-x.

16. Hsu RL, Kaye AD, Urman RD. Anesthetic Challenges in Robotic-assisted Urologic Surgery. Rev Urol. 2013;15(4):178-84. doi: 10.3909/riu0589.

17. Randell R, Honey S, Hindmarsh J, Alvarado N, Greenhalgh J, Pearman A, et al. A realist process evaluation of robot-assisted surgery: integration into routine practice and impacts on communication, collaboration and decision-making. Health Services and Delivery Research. 2017;5(20). doi: 10.3310/hsdr05200.

18. Randell R, Greenhalgh J, Hindmarsh J, Honey S, Pearman A, Alvarado N, et al. How do team experience and relationships shape new divisions of labour in robot-assisted surgery? A realist investigation. Health. 2019:19. doi: 10.1177/1363459319874115.

19. Raheem S, Ahmed YE, Hussein AA, Johnson A, Cavuoto L, May P, et al. Variability and interpretation of communication taxonomy during robot-assisted surgery: do we all speak the same language? BJU International. 2018;122(1):99-105. doi: 10.1111/bju.14150.

20. Higuchi T, Gettman M. Robotic Instrumentation, Personnel and Operating Room Setup. In: LM S, editor. Atlas of Robotic Urologic Surgery. Humana Press. 2011. p. 15-30. doi: 10.1007/978-1-60761-026-7_2.

21. Payne TN, Pitter MC. Robotic-assisted surgery for the community gynecologist: can it be adopted? Clin Obstet Gynecol. 2011;54(3):391-411. doi: 10.1097/GRF.0b013e31822b4998.

22. Mishra A, Catchpole K, McCulloch P. The Oxford NOTECHS System: reliability and validity of a tool for measuring teamwork behaviour in the operating theatre. Qual Saf Health Care. 2009;18(2):104-8. doi: 10.1136/qshc.2007.024760.

23. Robertson ER, Hadi M, Morgan LJ, Pickering SP, Collins G, New S, et al. Oxford NOTECHS II: a modified theatre team non-technical skills scoring system. PLoS One. 2014;9(3):e90320. doi: 10.1371/journal.pone.0090320.
